# MiR-182/Sestrin2 affects the function of asthmatic airway smooth muscle cells by the AMPK/mTOR pathway

**DOI:** 10.2478/jtim-2023-0108

**Published:** 2021-08-24

**Authors:** Yali Xiao, He Zhu, Jiahui Lei, Jing Xie, Ke Wu, Wenbo Gu, Jinxin Ma, Dongxue wei, Zhenhui Shu, Limin Zhao

**Affiliations:** Department of Respiratory and Critical Care Medicine, Zhengzhou University People’s Hospital, Henan Provincial People’s Hospital, Zhengzhou 450003, Henan Province, China; Department of Respiratory and Critical Care Medicine, Henan Provincial People’s Hospital, Zhengzhou 450003, Henan Province, China; Department of Respiratory and Critical Care Medicine, Henan University of Traditional Chinese Medicine, Henan Provincial People’s Hospital, Zhengzhou 450046, Henan Province, China; Department of Respiratory and Critical Care Medicine, Henan Provincial People’s Hospital, Zhengzhou University People’s Hospital, Henan University People’s Hospital, Zhengzhou 450003, Henan Province, China

**Keywords:** asthma, ASMCs, Sestrin2, miR-182, AMPK/mTOR pathway

## Abstract

**Background and Objectives:**

Asthma is a chronic inflammatory airway disease and brings heavy economic and spiritual burdens to patients’ families and the society. Airway smooth muscle cells (ASMCs) afect the development of asthma by secreting cytokines, growth factors, and prostates. The stress-inducing protein, Sestrin2, plays a vital role in antioxidant defense. The aim of this study is to investigate the role of Sestrin2 in asthma and its corresponding molecular mechanism.

**Materials and Methods:**

Airway remodeling was induced by construction of asthma rat model. Primary ASMCs were isolated through combining tissue block adherence and enzymatic digestion and identified by immunofluorescence staining. Gene expression was measured by quantitative real-time PCR (qPCR) and western blot (WB) experiments. Cell viability, proliferation, migration, and calcium flow of ASMCs were measured by Cell Counting Kit-8 (CCK-8), 5-ethynyl-deoxyuridine (EdU), Transwell, and Fluo-3AM, respectively. The binding of miR-182 and Sestrin2 3′-untranslated region (3′-UTR) was measured by luciferase reporter system and RNA-binding protein immunoprecipitation (RIP) analysis.

**Results:**

Sestrin2 expression was upregulated in asthma rat model and cell model. Overexpression of Sestrin2 enhanced the growth, migration, and calcium flow, and inversely, repression of Sestrin2 was reduced in ASMCs from the asthma group. MiR-182, one of the microRNAs (miRNAs) that possesses the potential to regulate Sestrin2, was downregulated in ASMCs from the asthma group. Further experiments revealed that Sestrin2 was inhibited by miR-182 and that overexpression of Sestrin2 reversed the miR-182–induced inhibition of the cellular progression of ASMCs from the asthma group. This study further investigated the downstream signaling pathway of Sestrin2 and found that increased expression of Sestrin2 activated 5′-adenosine monophosphate-activated protein kinase (AMPK), leading to the inactivation of mammalian target of rapamycin (mTOR) and thus promoting the growth, migration, and calcium flow of ASMCs from the asthma group.

**Conclusion:**

This study investigated the role of Sestrin2 for the first time and further dissected the regulatory factor of Sestrin2, ultimately elucidating the downstream signaling pathway of Sestrin2 in asthma, providing a novel pathway, and improving the understanding of the development and progression of asthma.

## Introduction

Asthma is a chronic airway inflammatory disease characterized by reversible airway obstruction and airway hyper-responsiveness and clinical symptoms, including coughing, wheezing, chest tightness, and dyspnea.^[1]^ It affects 1%–18% of the population in different countries,^[2]^ causing a heavy economic burden to the patients’ family and society. Airway remodeling is an important pathological change in asthma.^[3]^ Its typical structural changes include epithelial damage, mucus gland proliferation, basement membrane thickening, blood vessel regeneration, and airway smooth muscle cell (ASMC) hyperplasia and hypertrophy.^[4]^ Abnormal proliferation of ASMCs can lead to persistent structural damage.^[5]^ It is the most important cause of airway remodeling in asthma and the pathological basis of airflow limitation and airway hyper-responsiveness.^[3]^ Studies have shown that airway remodeling is related to the onset of severe asthma, which is one of the reasons why asthma patients are less responsive or even resistant to drugs used in conventional treatment.^[6, 7, 8]^ Therefore, exploration of the proliferation mechanism of ASMCs is a hotspot in the current research in airway remodeling of bronchial asthma, and looking for new targets to effectively prevent and treat the proliferation of ASMCs has become the key to the prevention and treatment of asthma.

Sestrin (sesn) proteins are a family of stress-inducing proteins that are highly conserved in evolution, and their expression is affected by cellular stress.^[9]^ Sesns are widely found in animals, and vertebrates have three sesn genes, namely, sesn1, sesn2, and sesn3. Among the three mammalian sesns (Sestrin1–3), sesn1 and sesn2 (also known as PA26 and Hi95) were initially identified as p53 target genes and sesn3 was identified as the forkhead winged helix transcription factor target gene (FOXO) family member.^[4]^ Sesn2 (SESN2, also known as Hi95) is an important member of the sesn protein family, which can be activated by a variety of metabolic conditions, such as hypoxia, DNA damage, oxidative stress, and endoplasmic reticulum stress (ERS).^[11]^ Studies have shown that activation of sesn2 expression plays an important role in reducing oxygen cluster (reactive oxygen species [ROS]) accumulation, maintaining energy balance, enhancing autophagy, reducing protein synthesis, slowing the progression of metabolic diseases, and regulating cell growth.^[5]^ Studies have shown that increased expression of Sestrin2 can inhibit inflammation and protect cardiomyocytes from damage.^[6]^ Early studies have shown that sesns are related to the regulation of human age-related diseases such as cancer, chronic inflammation, cardiovascular diseases, neuromuscular degenerative diseases, and metabolic-related diseases.^[7]^ Studies have shown that high expression of Sestrin2 *in vitro* can inhibit tumor cell proliferation.^[7]^ The role of sesn2 in inflammatory diseases and the immune system is poorly understood. Studies have confirmed that sesn2 can be expressed in M1 macrophages, and macrophages that highly express Sestrin2 have anti-inflammatory and repair effects.^[8]^ Furthermore, Sestrin2 significantly inhibits the mammalian target of rapamycin (mTOR) complex 1 (mTORC1) signal transduction of M1 macrophages, indicating that Sestrin2 has an important protective effect on the role of M1 macrophages in cardiac inflammation.^[8]^

A large number of studies have shown that activation of the 5′-adenosine monophosphate-activated protein kinase (AMPK)/mTOR pathway can regulate cell metabolism and inhibit cell proliferation in a variety of ways.^[9]^ A previous study demonstrated that activation of AMPK negatively regulates the activity of mTOR to inhibit the proliferation of ASMCs.^[10]^ Metformin, a widely used clinical treatment for type 2 diabetes, exerts anti-inflammatory and anti-proliferative effects by activating AMPK to inhibit tissue inflammation and tissue remodeling.^[11]^ Previous studies demonstrated that Sestrin2 played its role by modulating the AMPK/mTOR signaling pathway.^[12,13]^ Studies have found that Sestrin2 can control cell growth and metabolism by regulating the activity of the cellular AMPK/mTOR signaling pathway.^[14,15]^ Another study showed that Sestrin2 is a scaffold protein that mediates AMPK activation in the ischemic myocardium and interacts with the serine– threonine liver kinase B1 (LKB1), which is an upstream regulator of AMPK, to mediate AMPK activation in the ischemic myocardium, thereby inhibiting myocardial inflammation and protecting myocardial cells.^[16]^ However, whether Sestrin2 plays its role through modulating the AMPK/mTOR pathway in asthma is still unclear.

Therefore, the purpose of this study is to investigate the role of Sestrin2 in asthma and further dissect the regulatory and functional mechanisms of Sestrin2 in asthma. This study demonstrated that increased Sestrin2 expression in asthmatic rat and cell models, and that Sestrin2 promoted cell growth, migration, and calcium flow in asthmatic ASMCs. Furthermore, we investigated the regulation of Sestrin2 by miR-182 and ultimately verified that Sestrin2 exerted its function by affecting the AMPK/mTOR pathway in asthma, providing a novel signaling pathway, miR-182/Sestrin2/AMPK/mTOR, which might act as a potential diagnostic and therapeutic biomarker for asthma.

## Materials and Methods

### Model of airway remodeling in asthmatic rats

All experiments that were conducted in animals conformed to the ethical guidelines and were approved by the Research Ethics Committee of the Zhengzhou University. Twenty male Wistar rats (6 weeks old, 180–200 g body weight) were obtained from the Animal Experiment Center of Zhengzhou University and randomly divided into two groups: the control group (10 rats) and the asthma group (10 rats). One hundred milligrams of ovalbumin (OVA) antigen suspension (Med Chem Express, NJ, USA) and 1 mg of aluminum hydroxide (Med Chem Express) were intraperitoneally injected into the rats in the asthma group on days 1, 7, and 14. On day 21, the rats in the asthma group were administered atomized 2% OVA solution daily for 30 min for 6 weeks. Rats in the control group were intraperitoneally injected with phosphate-buffered saline (PBS) and subjected to atomized PBS for similar procedures. The animals were euthanized using chloral hydrate. The tracheal tissue was taken, washed with PBS, and fixed in 4% paraformaldehyde (PFA). H&E staining was commissioned by Wuhan Servicebio Biotechnology Co. Ltd. (Wuhan, China).

### Primary ASMC isolation and culture

Rats from the control and asthmatic groups were euthanized by intraperitoneal injection of 10% chloral hydrate. Tracheal smooth muscle was dissected and cut into 1 × 1 mm pieces under sterile conditions. Tissue samples were digested by an enzyme solution containing type I collagenase and trypsin (Invitrogen, USA) in a 37°C water bath for 30 min. Undigested tissues were removed by centrifugation, and the cells were cultured in Dulbecco's modified Eagle's medium (DMEM; HyClone, USA) containing 10% fetal bovine serum (FBS; HyClone), 0.1 mg/mL streptomycin, and 100 U/mL penicillin (Sangon Biotech, Shanghai, China) in an incubator with 5% CO_2_ at 37°C.

### Immunofluorescence analysis

The cells were washed with precooled PBS, fixed with 4% PFA dissolved in PBS, and permeabilized with 0.1% Triton X-100 dissolved in PBS. After blocking in bovine serum albumin (BSA) dissolved in PBS-Tween 20, the cells were probed with primary antibodies specific against α-smooth muscle actin (α-SMA) (1:50; Abcam, USA) at 4°C overnight, followed by incubation with Alexa 488 Fluor-conjugated IgG secondary antibodies (1:1000; Abcam). The nuclei were stained with Hoechst (Sangon Biotech). Images were recorded with a fluorescence microscope (Nikon, Tokyo, Japan).

### Gene regulation and transfection

Gene regulation of Sestrin2 was based on the lentivirus system. The ectopic expression clones of Sestrin2 were constructed based on the pcDH-puro plasmids (Lenti-Sestrin2), and small interfering RNA of Sestrin2 (siSestrin2) was obtained from Guangzhou RiboBio Co. Ltd. (Guangzhou, China). All plasmids were transferred into 293 cells using Lipofectamine-2000 reagent (Invitrogen) according to the manufacturer's protocol for 48 h, and the medium was harvested and added to the ASMCs to study the changes in gene expression.

MiR- 182 mimics, miR- 182 inhibitor, and their control small RNAs were obtained from Guangzhou RiboBio Co. Ltd. and transfected into cells using Lipofectamine-2000 for 48 h based on the manufacturer's protocol.

### RNA extraction and quantitative reverse transcription-PCR

The endothelial layer and the outer cortex of tracheal were removed, and the tracheal smooth muscle tissues were ground into white powder in liquid nitrogen. The cells were washed with PBS and harvested on ice. Total RNA was extracted using an RNA isolation kit (Junxin, Suzhou, China) based on the manufacturer's instructions. The purity of total RNA was determined using a NanoDrop Spectrophotometer (Thermo, USA). Five-hundred nanograms of total RNA was used for the synthesis of first-strand cDNA using the M-MLV Reverse Transcriptase Kit (Fermentas, USA) following the manufacturer's instructions. Gene expression was measured with the aid of the primers (GENEray Biotech, Shanghai, China) listed in Table 1 and the SYBR Green I-based qPCR detection kit (Junxin). A melting curve was established, and the PCR products were identified with a 1% agarose gel. The differences in gene expression were determined by the 2^−ΔΔCT^ method. The housekeeping gene β-actin was chosen as an internal control.

**Table 1:** Sequence of primers

Genes		Sequence
Rat-Sestrin2	Forward	5′-CGGTATCGCCAGTTCTCCTC-3′
	Reverse	5′-AGGTAAGAACACTGGTGGCG-3′
Rat-β-actin	Forward	5′-ATGGATGACGATATCGCTGC-3′
	Reverse	5′-CTTCTGACCCATACCCACCA-3′
Rat-miR-182	Forward	5′-CGGCGTTTGGCAATGGTAGAAC-3′
	RT	5′-GTCGTATCCAGTGCAGGGTCCGAGGTATTCGCACTGGATACGACAGAGTGTG-3′
Rat-U6	Forward	5′-CTCGCTTCGGCAGCACA-3′
	Reverse	5′-AACGCTTCACGAATTTGCGT-3′

### Western blotting

The endothelial layer and the outer cortex of tracheal were removed, and the tracheal smooth muscle tissues were ground into white powder in liquid nitrogen. The cells were washed with precooled PBS and harvested on ice. The samples were lysed with radioimmunoprecipitation assay (RIPA) lysis buffer (Sangon Biotech) supplemented with 1 mg/mL leupeptin (Sigma, China), and the concentration was determined using a bicinchoninic acid (BCA) kit (Junxin Biotech, Suzhou, China) based on the manufacturer's protocol. In total, protein extracts (20 μg) were loaded for electrophoresis on sodium dodecyl sulphate (SDS)-polyacrylamide gels (5%) and subsequently transferred to nitrocellulose membranes (Millipore, USA). The membranes were blocked with skimmed milk (5%) dissolved in TBS-Tween 20 buffer for 1 h at room temperature. The membranes were probed with primary antibodies against Sestrin2 (dilution of 1:1000, Ab 178518; Cambridge, UK), AMPK (dilution of 1:1000, Ab207442), *p*-AMPK (dilution of 1:1000, Ab133448), mTOR (dilution of 1:1000, CST2972; MA, USA), *p*-mTOR (dilution of 1:1000, CST5536), and β-actin (dilution of 1:3000; Proteintech, USA) at 4°C overnight, followed by incubation with HRP-conjugated IgG secondary antibodies (dilution of 1:3000; Proteintech, USA). The target genes were then developed using an enhanced chemiluminescence (ECL) kit (Junxin Biotech) following the manufacturer's instructions.

### Cell Counting Kit-8 analysis

Cell Counting Kit-8 (CCK-8) solution (10 μL; Junxin Biotech) was added to 96-well plates and kept in an incubator with 5% CO_2_ at 37°C for 2 h. The optical absorbance was detected at a wavelength of 450 nm using a spectrophotometer (Zuofei, Shanghai, China). Each experiment was performed in triplicate.

### 5-Ethynyl-deoxyuridine analysis

The cells were treated with 5-ethynyl-deoxyuridine (EdU; 200 μL; Junxin Biotech) and kept in an incubator with 5% CO_2_ at 37°C for 2 h. The cells were washed with precooled PBS, fixed with 4% PFA dissolved in PBS, permeabilized with 0.1% Triton X-100 dissolved in PBS, and stained with Apollo staining solution. The nuclei were stained with Hoechst (Sangon Biotech). Images were recorded with a fluorescence microscope (Nikon).

### Transwell analysis

The cells were washed with PBS, resuspended in DMEM without FBS, and plated into the inner Transwell chamber (Corning, USA). The 24-well plates were supplemented with DMEM supplemented with 10% FBS and kept in an incubator with 5% CO_2_ at 37°C overnight. The cells on the inner face of the Transwell chamber were removed. The cells on the upper face of the Transwell chamber were stained with 1% purple crystal and recorded with a fluorescence microscope (Nikon).

### Target prediction and luciferase reporter assay

TargetScan (www.targetscan.org) and microrna.org(http://www.microrna.org) were combined to predict the microRNAs (miRNAs) that might target the 3′-untranslated region (3′-UTR) sequences of Sestrin2 mRNA.

The 3′-UTR of Sestrin2 mRNA, which possesses the wild-type (Sestrin2-wt) or mutant (Sestrin2-mut) putative binding sites of miR-182, was cloned into the psiCheck-2 luciferase reporter plasmid. Sestrin2-wt and Sestrin2-mut were cotransfected with miR-182 mimics and mimic negative controls (NC) into ASMCs using Lipofectamine 2000 (Invitrogen) based on the manufacturer's protocol for 48 h. The luciferase activity was determined using a Luciferase Detection Kit (Junxin Biotech) following the manufacturer's instructions.

### RNA-binding protein immunoprecipitation analysis

The cells were washed with PBS and lysed by RIPA buffer and then, cells were incubated with Protein A/G beads (Biolinkedin biotechnology, Shanghai, China) at 4°C for 1 h. Then, they were centrifuged at 800 g for 1 min at 4°C. Take 50 μL supernatant from each sample as the input sample. To the remaining samples were added Protein A/G beads and Argonaute 2 (Ago 2) antibodies and incubated at 4°C overnight. Next day, the samples were centrifuged at 800 *g* for 1 min at 4°C and the supernatants were discarded. Then, the pellets were added to 1 mL Trizol and the RNA was extracted. The enrichment of miR-182 and Sestrin2 were measured by qPCR analysis.

### Statistical analysis

Statistical analyses were carried out using GraphPad Prism, version 5.03 (GraphPad Software, San Diego, CA, USA), and data are shown as the mean ± standard deviation. Student's *t*-test was performed in case of normal distribution and Kruskal–Wallis test in case of abnormal distribution. Statistical significance, which was defined as *P* < 0.05, was evaluated by Student's *t*-test.

## Results

### Upregulation of Sestrin2 in the airway and ASMCs of asthmatic rats

**Figure 1:**
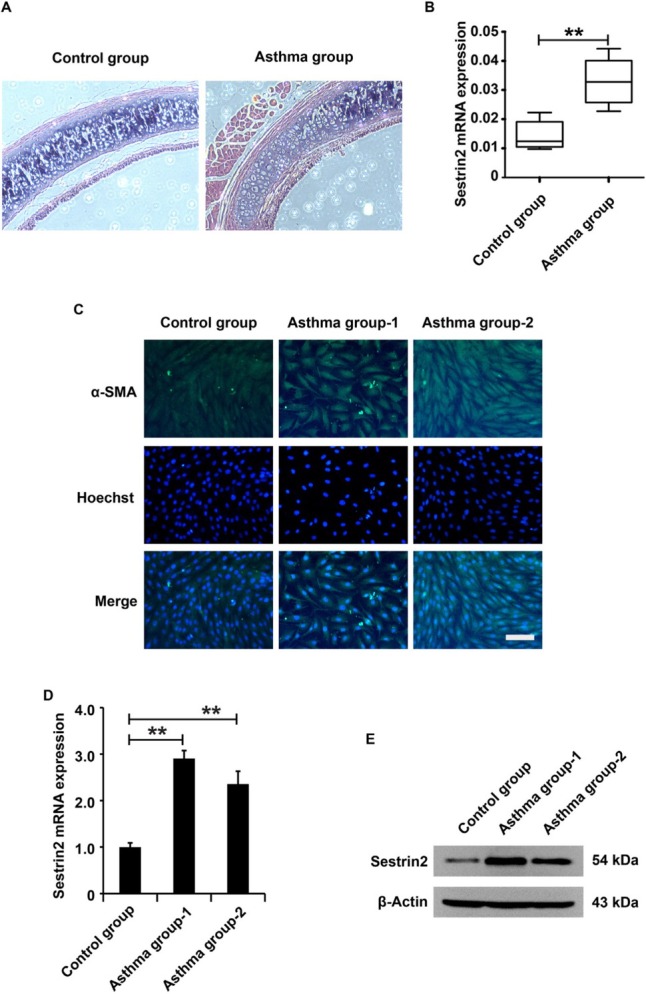
Upregulation of Sestrin2 in the airway of the asthma model. (A) HE assay analysis of the airway in an asthma rat model. (B) qPCR analysis of Sestrin2 expression in an asthma rat model. Ten rats in each group. (C) Immunofluorescence assay analysis of α-SMA expression in ASMCs. (D and E) qPCR and western blotting analysis of Sestrin2 expression in ASMCs. qPCR analysis was repeated three times. “Asthma group 1” represents the ASMCs isolated and cultured from the tracheal smooth muscle tissue of an asthmatic rat, while “Asthma group 2” represents the ASMCs isolated from another asthmatic rat. β-Actin served as an internal reference. ^**^P < 0.01.

To measure Sestrin2 expression in asthma *in vivo*, an asthma rat model was constructed. According to the pathological results, there is a thicker airway wall, basement membrane, and airway smooth muscle (ASM) layer in asthmatic rats than in control rats (Figure 1A), suggesting that an asthmatic rat model was successfully constructed in this study. Sestrin2 expression in rats from the asthma group was measured using quantitative reverse transcription-PCR (qRT-PCR) analysis. The data showed an increase in Sestrin2 expression in rats from the asthma group compared to the rats from the control group (Figure 1B). To determine Sestrin2 expression in asthma *in vitro*, primary ASMCs were isolated and immunofluorescence analysis was performed to identify the purity of primary ASMCs. According to the experimental results, almost all the cells were positive for α-SMA, which is a marker of smooth muscle cells (Figure 1C), suggesting that this study successfully isolated primary ASMCs. Next, Sestrin2 expression in ASMCs from the asthma group or control group was detected using qRT-PCR and western blotting analysis. The results displayed an increase in Sestrin2 expression in ASMCs from the asthma group compared to the ASMCs from the control group (Figure 1D and 1E), indicating that Sestrin2 might be involved in the progression of asthma.

### Sestrin2 promoted the cellular function of ASMCs from the asthma group

**Figure 2:**
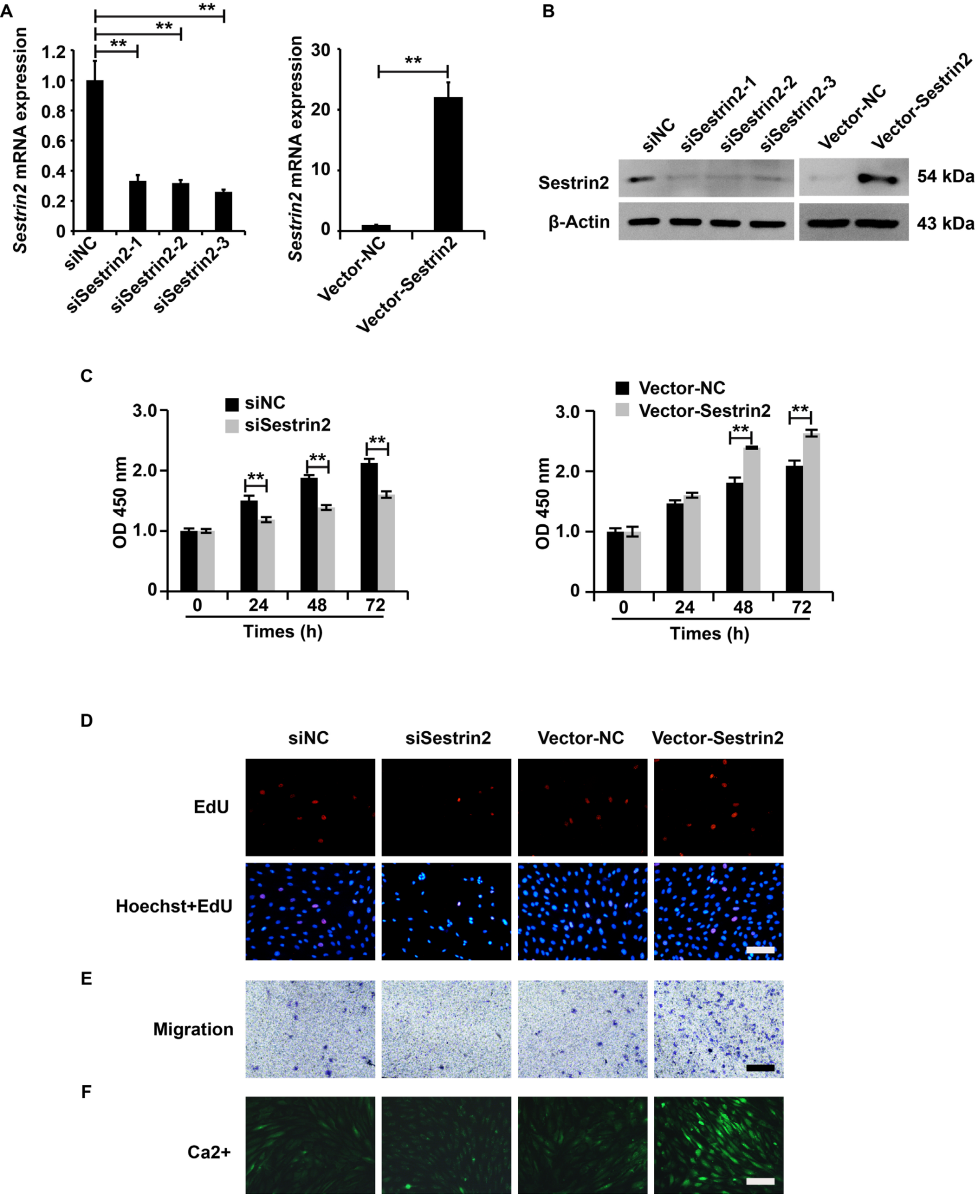
Sestrin2 promoted the cellular function of ASMCs from the asthma group. (A) qPCR analysis of Sestrin2 expression. qPCR analysis was repeated three times. (B) Western blotting analysis of Sestrin2 expression. (C) CCK-8 assay analysis of cell viability. qPCR analysis was repeated three times. (D) EdU assay analysis of cell proliferation. (E) Transwell assay analysis of cell migration. (F) Fluo-3AM assay analysis of calcium flow. β-Actin served as an internal reference. qPCR and CCK-8 analysis were repeated three times. ^**^P < 0.01.

The overexpression and downregulation system of Sestrin2 expression was constructed, and the efficiency of this system was assessed using qRT-PCR and western blotting analysis. Based on the results, we observed that Sestrin2 expression was significantly inhibited by Lenti-shSestrin2 and dramatically promoted by Lenti-Sestrin2 in ASMCs from the asthma group (Figure 2A and 2B), indicating that we successfully constructed an overexpression and downregulation system of Sestrin2 expression.

Then, we aimed to investigate the role of Sestrin2 in the cellular function of ASMCs from the asthma group. According to the results of the CCK-8, EdU, Transwell, and Fluo-3AM assays, the downregulation of Sestrin2 was inhibited, and the ectopic expression of Sestrin2 enhanced the cellular function, including the viability, proliferation, migration, and calcium flow, of ASMCs in the asthma group (Figure 2C–2F). Overall, these data revealed that Sestrin2 promoted the cellular function of ASMCs from the asthma group.

### Sestrin2 was regulated by miR-182 in ASMCs from the asthma group

**Figure 3:**
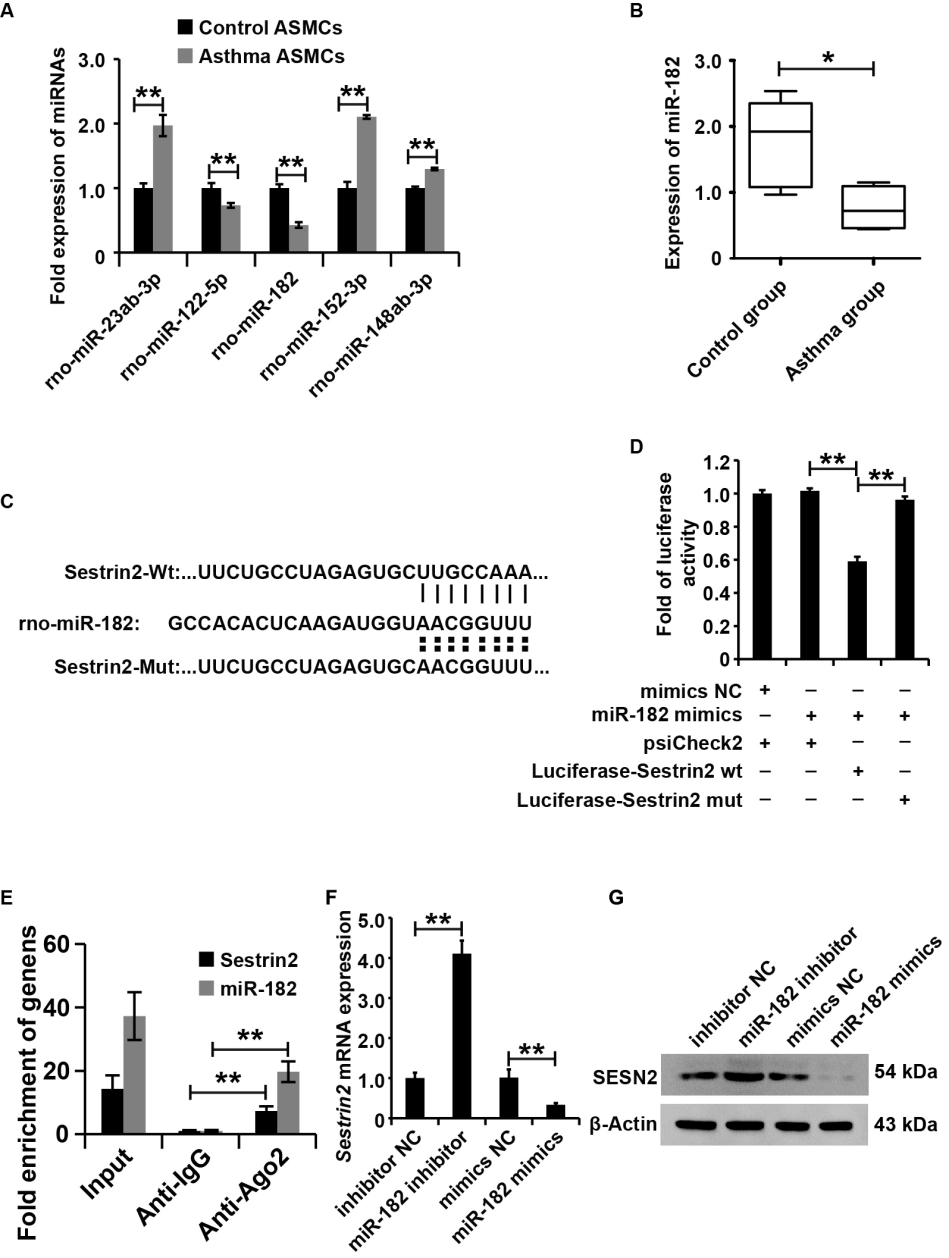
Sestrin2 was regulated by miR-182 in ASMCs from the asthma group. (A) qPCR assay analysis of miRNA expression in asthma and control ASMC cells. U6 served as an internal reference. (B) qPCR analysis of miR-182 expression in the air of asthmatic rats. U6 served as an internal reference. (C and D) Luciferase reporter assay analysis of the interaction between miR-182 and the 3'-UTR of Sestrin2. (E) Ago-RIP analysis of the interaction between miR-182 and Sestrin2. (F) qPCR analysis of Sestrin2 expression. (G) Western blot analysis of Sestrin2 expression. qPCR, luciferase reporter assay was repeated three times. miRNA expression was normalized to U6, and Sestrin2 expression was normalized to β-actin. *P < 0.05, **P < 0.01.

As noted earlier, Sestrin2 expression was upregulated in asthma both *in vivo* and *in vitro*. This study aimed to investigate the regulation of Sestrin2 expression in asthma. TargetScan (www. targetscan.org) and microrna.org (http://www.microrna.org) were combined to predict the miRNAs that might target the 3′-UTR sequences of Sestrin2 mRNA. According to the prediction results, several miRNAs, including miR-23ab, miR-122-5p, miR-182, miR-152-3p, and miR-148ab-3p, have the potential to regulate Sestrin2 expression. As shown in the qRT-PCR analysis data, miR-182 was significantly decreased in ASMCs from the asthma group compared to ASMCs from the control group (Figure 3A). The data in the rat model showed that miR-182 was dramatically lower in the airways of rats from the asthma group than in those from the control group (Figure 3B). Overall, these data revealed that miR-182, which is the candidate regulator of Sestrin2, was decreased in asthma both *in vitro* and *in vivo*.

To investigate whether miR-182 directly inhibited Sestrin2 expression in ASMCs, we cloned the 3′-UTR of Sestrin2 with wild-type (Luciferase-Sestrin2-wt) or mutant (Luciferase-Sestrin2-mut) miR-182–binding sites into luciferase reporter plasmids (Figure 3C) and detected the effect of the miR-182 mimics on luciferase activity. As shown in Figure 3D, miR-182 mimics inhibited the luciferase activity of ASMCs transfected with Luciferase-Sestrin2-wt, but no alteration of luciferase activity was observed in ASMCs transfected with Luciferase-Sestrin2-mut groups. To further confirm the interaction of Sestrin2 and miR-182, we performed the Ago-RIP to analysis of the binding among Ago, Sestrin2, and miR-182. The results demonstrated that Ago could recruit Sestrin2 and miR-182 (Figure 3E). Then, this study altered the miR-182 expression by transforming miR-182 mimics, mimic NC, miR-182 inhibitor, and inhibitor NC into ASCMs. According to the results of qRT-PCR and western blot analysis, miR-182 inhibited Sestrin2 expression in ASMCs (Figure 3F and 3G). Overall, these results indicated that miR-182 inhibited Sestrin2 expression in ASMCs.

### miR-182/Sestrin2 pathway affected the cellular function of ASMCs from the asthma group

**Figure 4:**
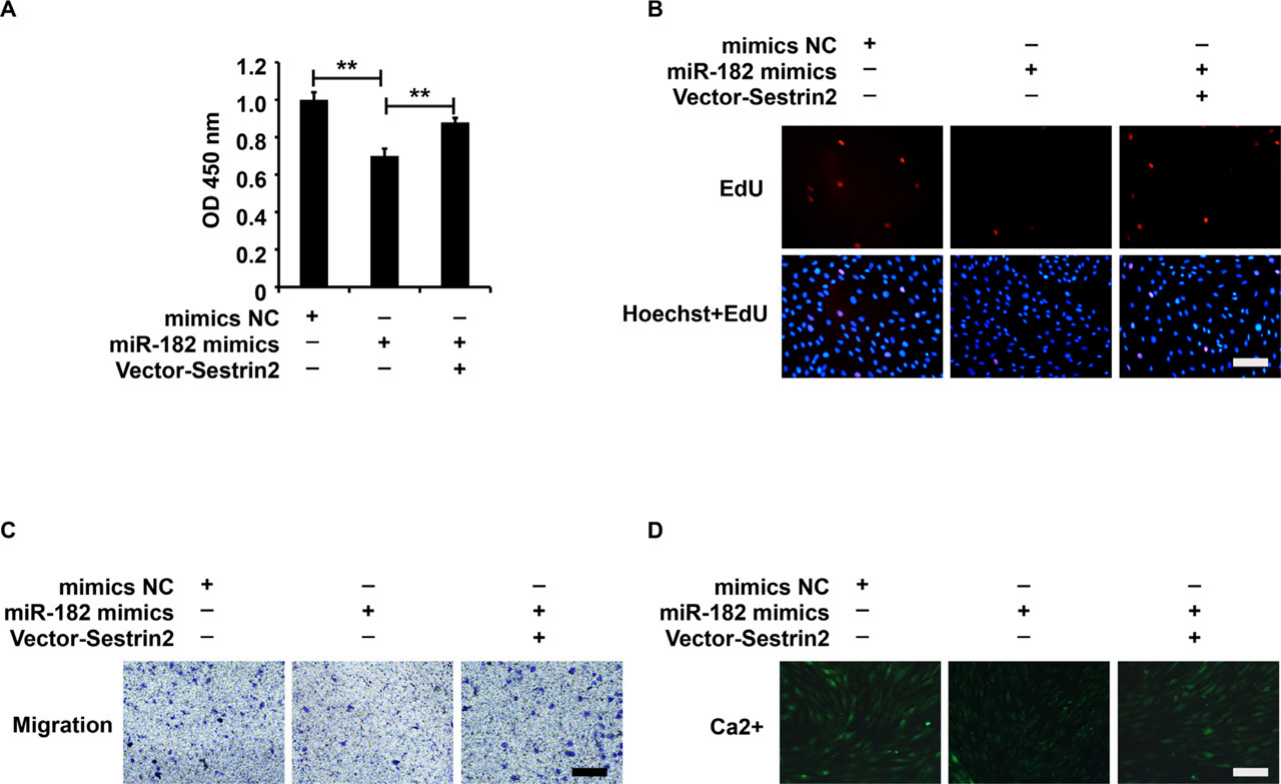
miR-182/Sestrin2 pathway affected the cellular function of ASMCs from the asthma group. (A) CCK-8 assay analysis of cell viability. (B) EdU assay analysis of cell proliferation. (C) Transwell assay analysis of cell migration. (D) Fluo-3AM assay analysis of calcium flow. CCK-8 analysis was repeated three times. ^**^P < 0.01.

The ASMCs were treated with the mimics NC+Lenti-NC, miR-182 mimics+Lenti-NC, or miR-182 mimics+Lenti-Sestrin2, and the cellular functions were measured using CCK-8, EdU, Transwell, and Fluo-3AM assays. Based on the data in Figure 4, Lenti-Sestrin2 reversed the cellular functions that were repressed by miR-182 mimics in ASMCs from the asthma group, indicating that miR-182 inhibited the cellular function of ASMCs from the asthma group by suppressing Sestrin2 expression.

### Sestrin2 activated the AMPK/mTOR signaling pathway in ASMCs from the asthma group

**Figure 5:**
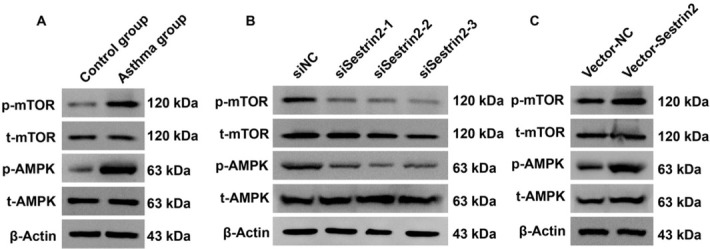
Sestrin2 activated the AMPK/mTOR signaling pathway in ASMCs from the asthma group. (A) Western blotting analysis of the AMPK/mTOR pathway in asthmatic and control ASMCs. (B and C) Western blotting analysis of the AMPK/mTOR pathway in ASMCs with altered Sestrin2 expression.

As noted previously, this study investigated the upstream regulatory factors of Sestrin2 and then aimed to study the downstream signaling pathway of Sestrin2. According to the results of western blotting analysis, the AMPK signaling pathway was inactivated and the mTOR signaling pathway was activated in ASMCs from the asthma group (Figure 5A). Then, Sestrin2 expression was modulated to investigate whether the AMPK/mTOR signaling pathway could be regulated by Sestrin2. Based on the results of western blot analysis, upregulation of Sestrin2 induced inactivation of AMPK and activation of the mTOR signaling pathway, while downregulation of Sestrin2 reversed these effects in ASMCs from the asthma group (Figure 5B and 5C). Collectively, these data suggested that Sestrin2 induced alterations in the AMPK/mTOR signaling pathway in ASMCs from the asthma group.

### Sestrin2 affected cellular function via activation of the AMPK/mTOR signaling pathway in ASMCs from the asthma group

**Figure 6:**
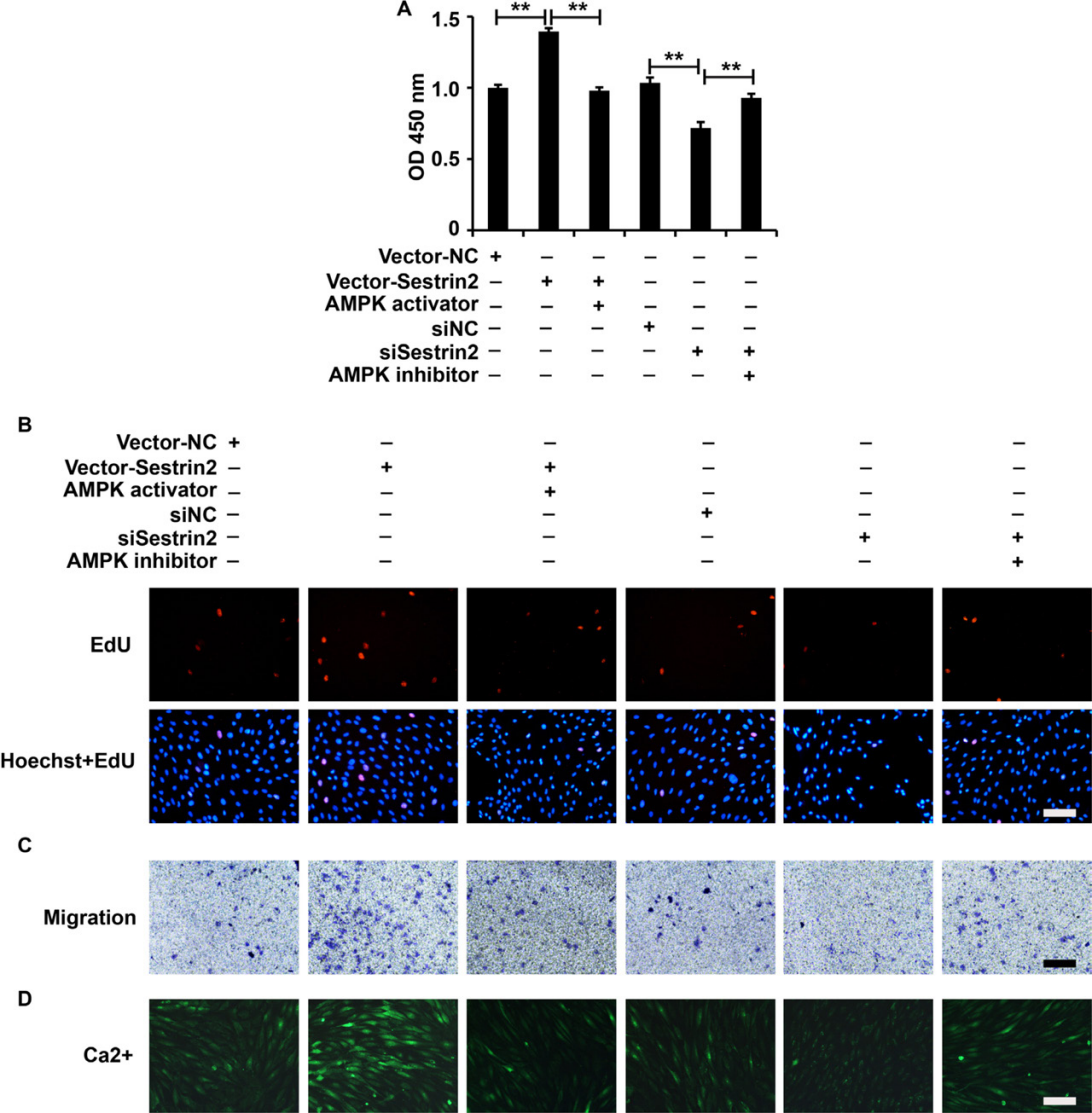
Modulation of the AMPK/mTOR signaling pathway affected the role of Sestrin2 in ASMCs from the asthma group. (A) CCK-8 assay analysis of cell viability. (B) EdU assay analysis of cell proliferation. (C) Transwell assay analysis of cell migration. (D) Fluo-3AM assay analysis of calcium flow. β-Actin served as an internal reference. CCK-8 analysis was repeated three times. ^**^P < 0.01.

To elucidate whether Sestrin2 played its role via the activation of the AMPK/mTOR signaling pathway in ASMCs from the asthma group, we overexpressed Sestrin2, followed by administration of an AMPK/mTOR inhibitor, or inhibited Sestrin2, followed by administration of an AMPK/mTOR activator in ASMCs from the asthma group. Based on the results of the CCK-8, EdU, Transwell, and Fluo-3AM assays, an AMPK/mTOR inhibitor inhibited the ectopic expression of Sestrin2 and an AMPK/mTOR activator attenuated the cellular function of silenced Sestrin2, including the viability, proliferation, migration, and calcium flow, in ASMCs in the asthma group (Figure 6A–6D). Collectively, these data revealed that Sestrin2 played its role via activation of the AMPK/mTOR signaling pathway in ASMCs from the asthma group.

## Discussion

As a widespread chronic inflammatory lung disease, asthma brings heavy economic and social burdens to patients’ families and the society.^[1]^ Features of asthma include chronic airflow obstruction and chronic airway inflammation and remodeling. ASM causes the airway to thicken and narrow through hyperplasia.^[17]^ ASM cells may also participate in chronic airway inflammation by expressing cytokines, growth factors, and proteases and induce phenotypic changes in ASM.^[18]^ In this study, a thicker airway wall and ASM layer was observed in the established asthma rat model compared to the control rats. In addition, the characteristics of overgrowth, enhanced migration ability, and enhanced calcium ion current were displayed in the ASMCs isolated from the airway of rats from the asthma model groups. This study showed that the ASMCs isolated from animal models of asthma still have overactivated cell biological functions.

As a family of stress-inducing proteins, Sestrins are highly conserved among species.^[19]^ Sestrins play important roles in homeostasis maintenance, cellular repair, and elimination of toxic metabolites formed by various injuries.^[19]^ Sestrins have three subtypes in mammals, including Sestrin1, Sestrin2, and Sestrin3. Also known as the p53 activation gene, Sestrin1 responds to stress, including ultraviolet light and g-rays, and is activated in a p53-dependent manner. ^[14]^ Sestrin2 is regulated by various damages, including hypoxia, oxidative stress, and DNA damage, and plays a vital role in antioxidant defense.^[5]^ Recently, Tsilogianni *et al*.^[20]^ first reported that Sestrin2 expression is upregulated in patients with severe asthma, indicating the involvement of Sestrin2 in the development and progression of asthma. Nevertheless, Tsilogianni *et al*.^[20]^ did not further study the function of Sestrin2 in animal models and cell models of asthma, nor did they study the regulation of Sestrin2 expression or the downstream regulatory signaling pathways of Sestrin2. This study revealed increased expression of Sestrin2 both in an asthma animal model and in a cell model and further elucidated the role of Sestrin2 in the cellular progression of ASMCs from a rat asthma model in which Sestrin2 enhanced cell growth, migration ability, and calcium ion current.

miRNAs are a well-studied class of encoding single-stranded RNA molecules with a length of approximately 22 nucleotides encoded by endogenous genes.^[21]^ miRNAs affect many kinds of cellular processes, regulating large numbers of genes by binding to the response elements in mRNA transcripts.^[22]^ Several studies have reported that Sestrin2 could be regulated by miRNAs. Hua *et al*.^[23]^ reported that Sestrin2 was regulated by miR-27a in human bladder cancer cells treated with ChlA-F. Kozak *et al*.^[24]^ showed that Sestrin2 was inhibited by the microRNA-200 family in endometrial cancer cell lines. Here, this study first systematically predicted that the miRNAs have a high potential to regulate Sestrin2 expression; it further tested these miRNAs in ASMCs from asthmatic rats and control rats and found that miR-182 had the lowest expression in ASMCs of asthmatic rats, which is the opposite of the expression of Sestrin2. Further research showed that Sestrin2 was regulated by miR-182 and that miR-182/Sestrin2 exerted an important role in the cellular progression of ASMCs from asthmatic rats. Thus, for the first time, our study elucidated the regulation of Sestrin2 by miR-182 in asthma.

As a highly conserved protein kinase, AMPK exists in all eukaryotic cells and is activated by phosphorylation of LKB1 kinase, and AMPK activation leads to mTORC1 inhibition.^[25]^ The AMPK/mTORC1 signaling pathway affects a variety of cellular processes.^[26]^ Sundararajan *et al*.^[27]^ reported that Sestrin2 induced the activation of AMPK and thus resulted in the inactivation of mTOR in monocyte activation. Shi *et al*.^[28]^ revealed that Sestrin2 afforded neuroprotection by activating AMPK and thus inactivating mTOR in neonatal hypoxic–ischemic encephalopathy. This study showed that the AMPK/mTOR pathway was modulated in asthma. Modulation of Sestrin2 affected activation of the AMPK/mTOR pathway in ASMCs, and further, modulating the AMPK/mTOR pathway affected the role of Sestrin2 in asthma.

This study identified the upregulation of Sestrin2 in an asthma rat model and cell model and revealed that the increased Sestrin2 promoted the growth, migration, and calcium flow of ASMCs in the asthma group, showed the regulation of Sestrin2 by miR-182, and elucidated the involvement of the AMPK/mTOR pathway in the role of Sestrin2 in ASMCs of asthma. Nevertheless, our research still has great shortcomings. First, our animal sample size is only 10, so we cannot rigorously demonstrate the argument of this study. We will increase the animal model sample size to verify the argument of this study in future work. Then, we need to comprehensively use mRNA expression profiling and other omics research methods to systematically screen the downstream target genes of Sestrin2 and further explore its function and molecular mechanism in the occurrence and development of asthma.

## Conclusion

This study identified the role of the increased expression of Sestrin2 in asthma and further dissected the upstream regulatory factor and the downstream regulatory signaling pathways of Sestrin2 in the cellular progression of ASMCs from the asthma group, providing a novel regulatory pathway in the development and progression of asthma.
